# Supplementary material for: Complexome profiling of the Chlamydomonas psb28 mutant reveals TEF5 as an early PSII assembly factor
Source: Plant Cell. 2025 Mar 17;37(6):koaf055. doi: 10.1093/plcell/koaf055 (PMC12164590; doi:10.1093/plcell/koaf055)
Supplement: koaf055_Supplementary_Data [file koaf055_supplementary_data.zip › psb28_interactive.zip!/plots/intensity/qualityPlots/peptides/_Cre01.g044400.t1.2.html]

### Cre01.g044400.t1.2

Trivial name:   
SeqCoverage [median]: 40.50  
Mass: 22.90 kDa  
  
 

| Sequence | MissedCleavage | Mass [Da] | wtIntensity | muIntensity | Score |
| --- | --- | --- | --- | --- | --- |
| ANVQTEASR | 0 | 974.48 | 2562.90 | 0.00 | 68.42 |
| DESGVSGDVK | 0 | 991.45 | 8980.84 | 17110.17 | 189.38 |
| DYDGHVFVK | 0 | 1078.51 | 2870.53 | 11148.17 | 129.47 |
| DYDGHVFVKELR | 1 | 1476.74 | 379.02 | 1437.37 | 36.45 |
| EAFRDLISIMD | 1 | 1308.64 | 830.01 | 1954.64 | 98.05 |
| IQARDESGVSGDVK | 1 | 1459.73 | 1574.27 | 4696.10 | 128.96 |
| LPGTLLLR | 0 | 881.57 | 0.00 | 6721.02 | 92.47 |
| SGGSEGGGLSGAER | 0 | 1219.54 | 6609.16 | 9554.17 | 155.99 |
| SGGSEGGGLSGAERAEVDELRR | 2 | 2188.05 | 1908.26 | 5212.11 | 92.99 |
| SGNWWEVR | 0 | 1032.48 | 0.00 | 495.34 | 64.27 |
|  |  |  | 25715.0 | 58329.1 |  |
